# Closing the Gap between Single Molecule and Bulk FRET Analysis of Nucleosomes

**DOI:** 10.1371/journal.pone.0057018

**Published:** 2013-04-18

**Authors:** Alexander Gansen, Aaron R. Hieb, Vera Böhm, Katalin Tóth, Jörg Langowski

**Affiliations:** 1 Department Biophysics of Macromolecules, DKFZ, Heidelberg, Germany; 2 Department of Chemistry, University of Washington, Seattle, Washington, United States of America; 3 Howard Hughes Medical Institute, Colorado State University, Fort Collins, Colorado, United States of America; Julius-Maximilians-University Würzburg, Germany

## Abstract

Nucleosome structure and stability affect genetic accessibility by altering the local chromatin morphology. Recent FRET experiments on nucleosomes have given valuable insight into the structural transformations they can adopt. Yet, even if performed under seemingly identical conditions, experiments performed in bulk and at the single molecule level have given mixed answers due to the limitations of each technique. To compare such experiments, however, they must be performed under identical conditions. Here we develop an experimental framework that overcomes the conventional limitations of each method: single molecule FRET experiments are carried out at bulk concentrations by adding unlabeled nucleosomes, while bulk FRET experiments are performed in microplates at concentrations near those used for single molecule detection. Additionally, the microplate can probe many conditions simultaneously before expending valuable instrument time for single molecule experiments. We highlight this experimental strategy by exploring the role of selective acetylation of histone H3 on nucleosome structure and stability; in bulk, H3-acetylated nucleosomes were significantly less stable than non-acetylated nucleosomes. Single molecule FRET analysis further revealed that acetylation of histone H3 promoted the formation of an additional conformational state, which is suppressed at higher nucleosome concentrations and which could be an important structural intermediate in nucleosome regulation.

## Introduction

The nucleosome is the basic repeating unit of chromatin. It regulates DNA accessibility, and its structural variability has profound influence on genetic function. The nucleosome consists of approximately 150 bp of DNA wrapped around a histone protein octamer containing two copies of each of the histones H2A, H2B, H3 and H4 [1]. The string of nucleosomes is further organized into the chromatin fiber and higher order structures. Structural changes in nucleosomes alter the local chromatin morphology, which modulates the accessibility of DNA to nuclear proteins such as transcription factors or polymerases. To understand the complex role of chromatin structure in gene regulation, we first need to elucidate the structural transitions that occur within nucleosomes.

Since the discovery of nucleosomes in the early 1970′s [2], many biophysical studies have characterized the shape and size of single nucleosome particles and nucleosome arrays [3], [4]. Later, X-ray crystallography gave us atomic resolution of the compacted mononucleosome [5]. Yet, despite intensive research, little is known about the dynamic properties of the nucleosome. However, recently fluorescence resonance energy transfer (FRET) [6], [7] has proven a useful tool for exploring nucleosome dynamics. FRET is the distance-dependent energy transfer between a donor and an acceptor fluorophore that are attached to DNA and/or protein in a macromolecular complex; changes in architecture can then be observed via changes in the interfluorophore distance. FRET has been used to follow changes in nucleosome structure induced by spontaneous linker DNA dynamics [8]–[10], nucleosome remodeling [11], [12], changes in DNA sequence [13], [14], histone modification and content [14]–[18], DNA modifications [19] or nucleosome disassembly and reassembly [20], [21]. Many of these structural changes are linked to changes in nucleosome dynamics, stability and, ultimately, genetic function.

**Figure 1:**
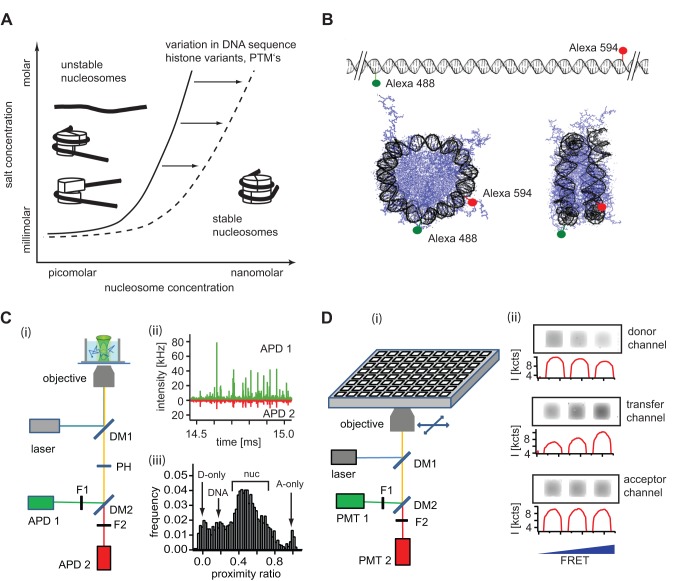
A combined single molecule – bulk FRET approach to study nucleosome stability. **A**) Theoretical diagram of nucleosome stability as a function of salt and nucleosome concentration (adapted from ref. [22]). The solid line represents the amount of salt needed to destabilize nucleosomes at a given nucleosome concentration. Nucleosomes generally remain stable at higher concentrations and lower ionic strength, dissociation occurs at elevated ionic strength and nucleosome concentrations in the sub-nM range. The dashed line represents changes in nucleosome stability from altered nucleosome composition. **B)** DNA labeling for nucleosome FRET experiments. 170 bp long DNA fragments were labeled at positions -42 and +52 from the dyad axis. In the intact nucleosome both dyes are located ≈ 6 nm apart, allowing for FRET, while in a fully dissociated nucleosome or free DNA fragment both dyes are too far apart to undergo FRET. **C**) (i) Schematic of confocal single molecule detection of nucleosomes in solution. A detailed description of the setup is given in Section S1 in File S1. (ii) The passage of individual nucleosomes through the focus generates bursts of fluorescence. (iii) For each burst a proximity ratio is calculated and data binned for histogram analysis. The position of relevant subpopulations in the histogram is indicated. **D**) (i) Schematic setup for microplate-scanning FRET (μpsFRET). Samples are loaded into a 384-well multiplate and imaged in three spectral channels using a commercial Typhoon™ multimode scanner with confocal optics (i). Grey scale images and intensity profiles of samples with different bulk FRET efficiencies (ii). Higher FRET leads to a decrease of signal in the donor channel and a corresponding increase of signal in the transfer channel. The signal in the acceptor channel remains unaffected. From these intensities P-values are calculated for each well. Abbreviations: DM: dichroic mirror, F: emission filter, APD: avalanche photodiode, PMT, photomultiplier tube, PH: pinhole.

Nucleosome stability is frequently regulated through changes in composition, e.g. DNA sequence, posttranslational modifications (PTMs) and histone variants. *In vitro* assays that probe the role of these modifications on nucleosome structure often rely on varying ionic strength and sample concentration to induce measurable changes. Nucleosomes are generally stable at low ionic strength and high nucleosome concentrations, while elevated ionic strength (>300 mM) or dilute concentrations (<1 nM) promote dissociation, see Fig. 1A (adapted from ref. [22]). Also, changes in nucleosome composition can alter the stability-defining properties that depend on salt and nucleosome concentration [23], [24]. To measure a wide range of stability-defining conditions, experiments must be performed over a broad range of sample concentrations from low picomolar to high nanomolar [25], [26]. Currently, no intensity-based FRET-based experiment can probe this wide a concentration range with equal sensitivity. Bulk methods become insensitive at low nanomolar concentrations, need rather large sample amounts and cannot discriminate intermediate states within a heterogeneous ensemble. The latter can be achieved with diffusion-based single molecule FRET experiments (smFRET), but these experiments are generally limited to concentrations <100 pM and are very time consuming. Our understanding of nucleosome structure and stability would benefit from an experimental strategy that can efficiently probe structural heterogeneity at arbitrary sample concentrations.

By combining our previously described extension of nucleosome smFRET to bulk concentrations (“quasi-bulk smFRET” [14]) with an innovative bulk FRET assay that is sensitive down to concentrations near those used for single molecule spectroscopy, we have developed such a strategy. Quasi-bulk smFRET can, in principle, probe the structural heterogeneity under arbitrary conditions: however, in order to find those conditions where relevant structural changes occur, one would have to screen many different samples through smFRET, which is extremely time consuming. Therefore, a strategy is needed to rapidly screen for suitable conditions in bulk first, before detailed single molecule experiments are performed. Such a bulk assay needs to be sensitive enough to detect sub-nanomolar concentrations and fast enough to screen many samples in a short time. By measuring FRET in 384-well microplates [27] and laser induced fluorescence detection, we can meet both requirements and efficiently explore nucleosome stability over a wide range of conditions. This “microplate-scanning FRET” (μpsFRET) methodology is sensitive to concentrations below 150 pM, consumes small amounts of sample and improves sample throughput compared to conventional bulk assays. Using this scheme, we rapidly screened for changes in nucleosome structure and stability upon acetylation of histone H3 under both bulk and single molecule conditions. We found evidence for an intermediate nucleosome conformation that exists prior to gross unwrapping and which is promoted by acetylation of histone H3.

## Materials and Methods

### a) Preparation of labeled mononucleosomes

Fluorescently labeled 170 bp DNA fragments centered on the 601 nucleosome positioning element [28] or the natural 5S rDNA sequence were prepared by PCR as described previously [14], [15]. Donor and acceptor labels were placed at positions −42 and +52 with respect to the dyad axis via an amino-C6 linker (see Figure 1B). For successful FRET experiments, in particular in an ensemble format, the amount of single labeled DNA has to be minimized. The quality of the labeled primers was first checked on a native polyacrylamide gel. After PCR, labeled DNA fragments were purified on an ion exchange column (Waters) using HPLC (Unicam); only the fraction with best ratio between Alexa594 and Alexa488 absorption was used for subsequent nucleosome reconstitution. We estimated the fraction of non double-labeled DNA after purification to be less than 5%.

Individual histones were expressed and purified as described previously [29]. Where needed, histone H3 was chemically treated using acetyl phosphate, resulting in random acetylation of the lysines [15]. DNA fragments were mixed with histone octamers at 2 M NaCl-TE buffer and reconstituted into nucleosomes by gradual salt dialysis down to 5 mM NaCl. The molar ratio between DNA and octamer was optimized between 1∶1.3 and 1∶2 to avoid aggregation and to minimize excess free DNA. Where needed, nucleosomes were centrifuged at 10,000 rpm (Eppendorf Centrifuge 5417R, corresponding to an rcf of 10600 g) for 10 minutes to remove residual aggregates. The quality of nucleosomes was checked by native PAGE; samples containing more than 15% free DNA were excluded from further analysis. Nucleosomes were stored in stock solution at 4 °C for up to 2 weeks.

### b) Confocal single molecule experiments

For smFRET experiments, nucleosomes were freshly diluted into the experimental buffer; TE buffer, pH 7.5, supplemented with 0.01% Nonidet P40 (Roche Diagnostics), 0.5 mM ascorbic acid to minimize photobleaching, and NaCl as noted. Approximately 40 pM labeled nucleosomes were mixed with an appropriate excess of unlabeled nucleosomes that were reconstituted with unlabeled, 170 bp long DNA fragments. smFRET data were recorded on a homebuilt confocal system [14], which is described in Section S1 in File S1. A schematic view of the device is shown in Figure 1C. All confocal experiments were performed in 384-well microplates (SensoPlate Plus, Greiner Bio-One). smFRET data were analyzed by our own software which filtered the raw data and selected single molecule events from the data stream provided by a time-correlated-single-photon-counting board (TimeHarp200, PicoQuant). A burst was defined as a group of at least 50 photons with a mutual separation of less than 120 µs. Single molecule proximity ratio histograms were built from selected single particle events and further analyzed with IGOR Pro software (WaveMetrics).

### c) Plate scanning FRET analysis

A variable mode scanner (Typhoon 9400, GE Healthcare) was used to measure the proximity ratio of samples incubated in 384-well microplates. Figure 1D shows a schematic view of the setup. A confocal laser spot with a diameter of a few μm (the exact operation parameters were not provided by the manufacturer) was rapidly scanned over the sample. All images were acquired with a pixel resolution of 100 μm with the image plane set to a height of 3 mm above the scanner surface. This placed the focus inside the microplate chambers. Fluorescence was recorded on two photomultiplier tubes (PMT) with voltages set between 600 V and 700 V. Fluorescence images were acquired in three spectral windows (donor channel: excitation at 488 nm, detection at 500–540 nm; acceptor channel: excitation at 532 nm, detection at 595–625 nm; transfer channel: excitation at 488 nm, detection at 595–625 nm). Images were analyzed with ImageQuant™ software and proximity ratios were calculated for each well as described below.

Prior to use, the microplates were cleaned by soaking in 1% Hellmanex solution (Hellma) for 30 minutes twice, with thorough washing with ddH_2_O in between. The wells were then treated with 100 mM HCl for 30 minutes and cleaned with ddH_2_O. After repeating the acid treatment at least once, microplates were dried under low vacuum. To passivate the surface, each well was filled with Sigmacote^™^ solution, incubated for 15–20 seconds and washed with ddH_2_O. The plates were again dried under low vacuum and sealed with film (Bio-Rad) to avoid exposure to dust and stored for subsequent use.

### d) Estimation of FRET efficiencies via the proximity ratio

Energy transfer was estimated from the sensitized emission of the acceptor upon selective donor excitation [7]. Fluorescence was detected in two spectral windows, yielding signal intensities I_D_^0^ and I_T_^0^ for the donor and transfer channel, respectively. Depending on the type of the experiment; these represent either the intensity within a region of an image (μpsFRET) or the number of donor and acceptor photons per single molecule burst (smFRET). Intensities were corrected for background from the buffer solution (B_D_ and B_T_), spectral crosstalk from the donor into the transfer channel (α_DT_) and direct excitation of the acceptor dye (f_dir_), yielding corrected intensities
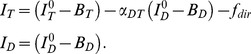
(1)


All correction factors were determined in independent control experiments as described in Section S1 in File S1. The proximity ratio P was calculated as a measure of energy transfer:
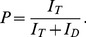
(2)


## Results

To bridge the gap between single molecule and bulk experiments we need to extend them to concentrations beyond their traditional limits. We first review our approach to single molecule detection at nanomolar sample concentrations and beyond. We then describe the microplate bulk FRET assay which allows us to obtain reliable FRET efficiencies from many samples in parallel and at concentrations as low as 100–150 pM. We conclude with a demonstration of how both techniques can be used to analyze structural changes in nucleosomes induced by acetylation of histone H3.

### a) Measuring single molecule FRET under bulk concentrations

**Figure 2:**
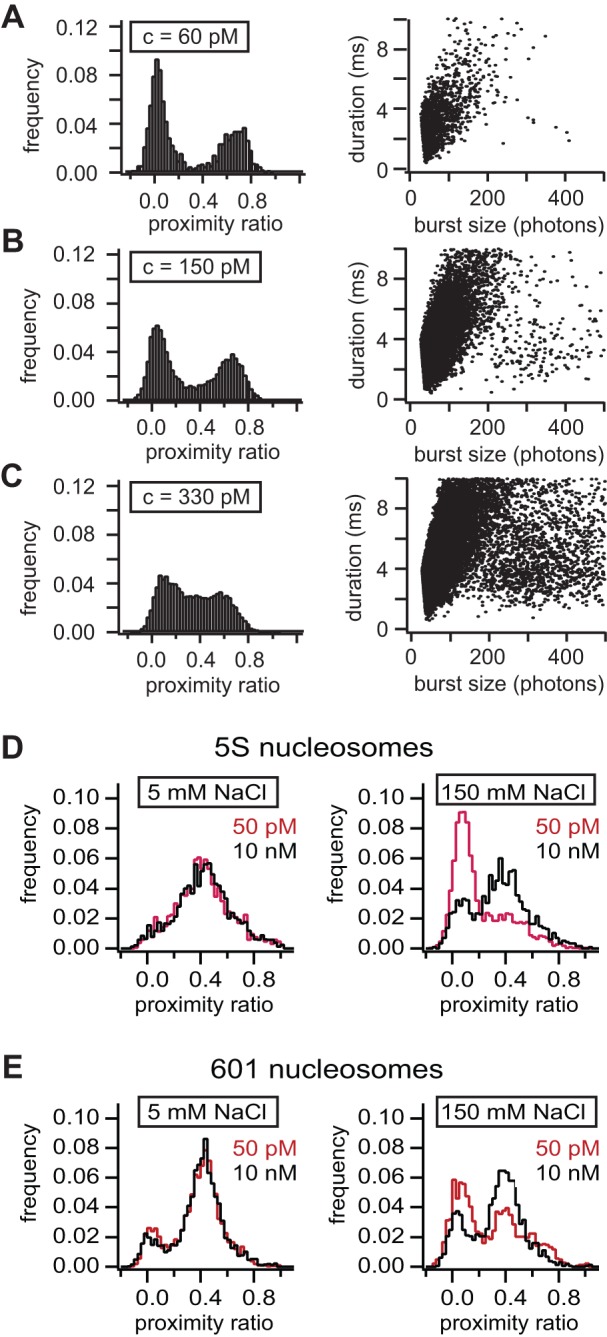
Working range of conventional and quasi-bulk single particle FRET. **A**–**C**) smFRET histograms and burst size to burst duration distributions for a binary DNA mixture (noFRET and FRET-active) at 60 pM (A), 150 pM (B), and 330 pM (C) sample concentrations. While at 60 pM both subpopulations are clearly separated, coincident detection of both species occurs at 150 pM and above. The presence of multi-particle events is evident from the burst size to burst duration distribution. While at 50 pM burst duration and burst size strongly correlate, additional populations appear outside the ellipsoidal point cloud at higher sample concentrations. **D, E**) Principle of quasi-bulk smFRET of nucleosomes. Nucleosomes were reconstituted on 5S rDNA (D) or the high affinity Widom 601 sequence (E). Histograms are shown for 5 mM or 150 mM salt concentrations and in the presence or absence of 10 nM unlabeled nucleosomes. At 5 mM NaCl (left panels) most nucleosomes were intact as expected from Figure 1A. At 150 mM NaCl (right panels) and in the absence of unlabeled nucleosomes, diluted nucleosomes dissociated, whereas under quasi-bulk conditions, nucleosomes on both 5S and 601 DNA remained intact.

Confocal single molecule FRET is highly sensitive and efficient for probing conformational dynamics of biomolecules. It analyzes individual particles as they diffuse through a small (<1 fL) observation volume. Since the ensemble is probed one molecule at a time, conformational subspecies can be resolved in a histogram of the measured energy transfer or proximity ratio (see Figure 1C). This feature, however, comes at the expense of limited sample throughput and rather long acquisition times. Furthermore, the useable range of concentrations and time scales are quite limited. In principle, infinitely low sample concentrations could be detected, but experiments with sample concentrations below a few pM take too long for most applications. At sample concentrations above 100 pM (depending on the optical setup) the simultaneous presence of more than one particle in the focus is no longer negligible. For illustration, Figures 2A–C show histograms from a mixture of two different DNA constructs at successively higher concentrations, one with zero energy transfer and one with the fluorophores close enough to permit FRET. At 50 pM, the peaks in the histogram are well separated, while samples containing 150 pM DNA and more show a broadened and less defined distribution due to coincident detection of the two species in one burst. A simple way to visualize the presence of such multi-particle events is to plot the number of photons detected per burst against burst duration [30]. At lower concentrations, both parameters strongly correlate, indicating that the majority of bursts are single molecule events; a longer presence in the focus results in proportionally more photons being emitted. At high sample concentrations, additional events are found outside the ellipsoidal correlation zone; either photons are detected at a higher rate than expected or the events last much longer than expected. Each indicates the simultaneous presence of multiple molecules in the focus, when smFRET experiments no longer reflect the true heterogeneity in the ensemble.

This limited working range severely restricts the usefulness of standard smFRET assays for the analysis of nucleosome structure. Nucleosome stability depends on DNA sequence: the dissociation constant of one of the strongest natural nucleosome positioning sequences, the 5 S rDNA, was previously estimated to 30–60 pM at 50 mM NaCl [25], which is still amenable to smFRET. Higher salt, however, will promote rapid dissociation under these concentrations. Weaker nucleosome positioning sequences will show dissociation constants of several hundred pM or more at physiological salt; impeding detailed smFRET analysis. To date, most diffusion-based smFRET experiments have been performed only on the strongest nucleosome positioning sequence, the artificial “Widom 601”, with at least a 100-fold higher affinity to the octamer than 5S rDNA [31], [32].

Most nucleosomes are too unstable to be probed at picomolar concentrations and an alternative method is needed to overcome these limitations. One often used approach is to add an excess of unlabeled complexes to raise the overall concentration to bulk levels. We have recently shown that nucleosome dissociation at dilute concentrations can be suppressed by the addition of native nucleosomes isolated from HeLa cells [14]. This concept was then refined into a quasi-bulk smFRET strategy to induce dilution-driven dissociation in smFRET by adjusting the total nucleosome concentration with unlabeled reconstituted nucleosomes [20].

Single molecule signals arise from fluorescently labeled particles diffusing through the focus; thus, unlabeled particles will not be observed and do not contribute to the distribution function. Doping the total nucleosome concentration with a tiny fraction (<<100 pM) of labeled molecules ensures single molecule discrimination, and we can make a large range of nucleosome concentrations accessible to single molecule studies. While we still observe fluorescent molecules one-at-a-time, the overall nucleosome concentration is much larger than the observed concentration. The structural heterogeneity under bulk conditions is now reflected in the labeled subset under the reasonable assumption that labeled and unlabeled nucleosomes behave identically.

Quasi-bulk smFRET is illustrated in Figures 2D and 2E, which show smFRET histograms of nucleosomes reconstituted on two positioning sequences of equal length and identical labeling, the highly stable “Widom 601” and the weaker positioning 5 S rDNA. First, 50 pM labeled nucleosomes were incubated with and without 10 nM unlabeled nucleosomes at 5 mM NaCl for 30 minutes and analyzed. As we would expect from Figure 1A, most nucleosomes were found in the stable conformation regardless of the total nucleosome concentration; histograms at 50 pM and 10 nM nucleosome concentrations were indistinguishable. We next incubated 50 pM labeled nucleosomes at 150 mM NaCl and measured smFRET histograms after 30 minutes. The less stable 5S sample shows a significant loss of FRET-active complexes, whereas the 601 sequence shows only a small decrease in FRET species. If the same amount of fluorescent nucleosomes was incubated with an excess of 10 nM unlabeled nucleosomes, however, the majority of both 5 S and 601 nucleosomes remained intact. Previous bulk experiments have shown that nucleosomes dissociate at significantly higher ionic strength when nucleosome concentrations are increased to nanomolar concentrations [25]; therefore quasi-bulk smFRET experiments reflect the behavior of the total ensemble and not only of the subset of labeled nucleosomes.

### b) Measuring ensemble FRET near single molecule conditions

While we have shown that it is possible to achieve single molecule sensitivity at high sample concentrations, such experiments usually take tens of minutes to build a statistically reliable histogram; see Figure S1. Considering that DNA sequence and modifications can dramatically affect nucleosome stability, a single molecule study would require many experiments to determine the appropriate conditions for testing the structural changes one is interested in. The whole study quickly becomes extremely time consuming. Standard fluorometry is often used to analyze multiple experimental conditions. While fairly dilute samples (<1 nM) can be analyzed fluorometric FRET spectroscopy requires long acquisition times (minutes) and probes only one condition at a time. Furthermore, one needs to ensure that the parameters of the optical setup, as well as sample handling and incubation, are identical throughout the test series. These challenges of bulk fluorometry warrant the exploration of alternative FRET methods that a) provide a fast standard assay, b) require small amounts of sample only and c) can analyze multiple samples at the same time.

To do so, we have adapted a bulk FRET assay based upon 384-well microplates [27], to enhance the performance of FRET experiments at very low sample concentrations. We refer to this method as “microplate-scanning FRET” (μpsFRET), since a commercial multimode scanner is used to image the fluorescence from a section of a microplate that is filled with the samples. For each well, the proximity ratio is calculated individually. Dye-specific laser excitation and detection by photomultiplier tubes allow working with much less sample to achieve sufficient signal strength. Scanning of the laser beam over an extended area and compartmentalization of samples on a microplate ensure fast analysis of many samples at the same time, with identical optical settings and incubation periods.

At low nanomolar concentrations and below, interactions of the sample with the walls of the experimental chamber become significant. We have observed that over time, nucleosomes destabilize as histones adsorb to the container walls during the experiment (see Figure S2). This reduces the histone concentration in solution, further destabilizing the nucleosomes. To reduce adsorption to the microplate surface, we have tested various passivation strategies to prevent time-dependent nucleosome destabilization. Passivation of each chamber with Sigmacote™ and the addition of a small amount of detergent (0.01% Nonidet P40) into the buffer solution was found to be optimal for our experiments.

**Figure 3:**
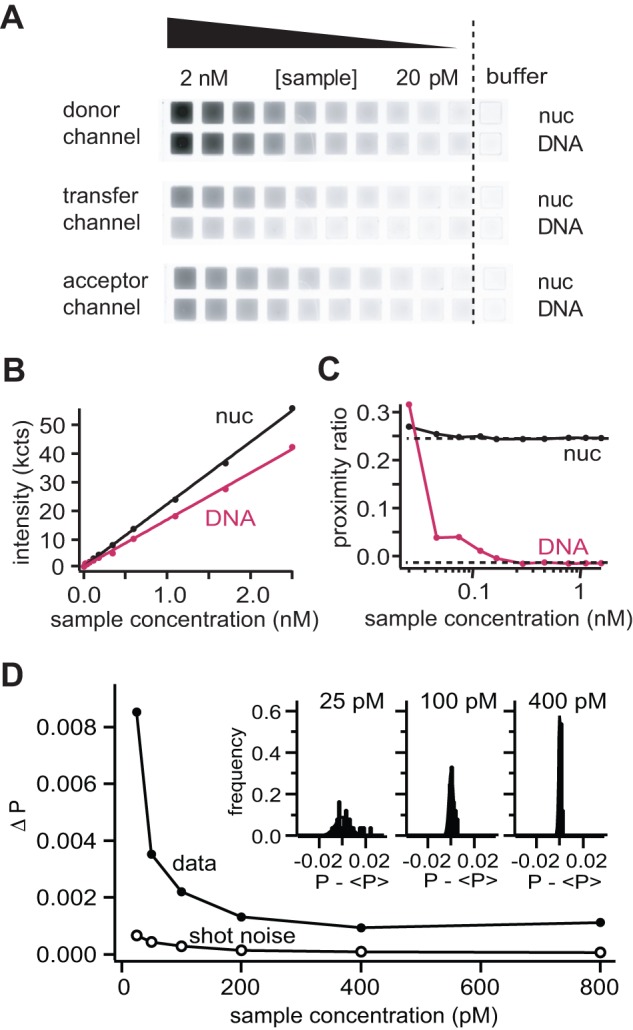
Characterization of microplate-scanning FRET spectroscopy (μpsFRET). **A)** μpsFRET grey scale images of a nucleosome sample (nuc) and a DNA fragment (DNA) at different sample concentrations (donor channel: excitation at 488 nm, detection at 500–540 nm; transfer channel: excitation at 488 nm, detection at 595–625 nm; acceptor channel: excitation at 532 nm, detection at 595–625 nm). Due to the absence of FRET, the DNA sample has a lower signal in the transfer channel. Concentrations are (from left to right): 2.5 nM, 1.7 nM, 1.1 nM, 600 pM, 350 pM, 180 pM, 120 pM, 70 p M, 40 pM, 20 pM, The last row to the right contained pure buffer solution. **B**) A plot showing the integrated fluorescence signal (donor channel + transfer channel) as a function of sample concentration. The measured intensity is linear throughout the dilution series. Concentrations below 50 pM can still be distinguished from background. **C**) A plot showing calculated P-values of nucleosomes and DNA as a function of sample concentration. For both samples P-values were consistent at larger concentrations, while for DNA P deviated at concentrations lower than 200 pM. Nucleosomal P-values were consistent to slightly lower concentrations (100 pM). **D**) Noise analysis of P-values from a donor-only sample under sub-nanomolar concentrations. Black circles are experimental standard deviations from 25 wells, white circles show estimated shot noise values. The low signal to noise level at lowest concentrations results in a large well-to-well variation in P. Shot noise accounts for <15% of the total uncertainty only, showing that the major source of uncertainty Is of different origin. The insert figures show well-wise P-distributions for 25 pM, 100 pM and 400 pM fluorophore.

To understand the limits of μpsFRET, we first determined its sensitivity and resolution. Figure 3A shows grey-scale images of all three detection channels for nucleosomes and free DNA with concentrations ranging from 2.5 nM to 20 pM. Low salt concentration (10 mM NaCl) was used to avoid dissociation. The summed intensity in the donor and transfer channel is proportional to sample concentration throughout the concentration range (Figure 3B), demonstrating proper sample integrity; concentrations as low as 50 pM are easily discriminated from background. This sensitivity limit is within the concentration range used in standard smFRET experiments and thus links this method to smFRET.

While a single labeled species can be detected with high sensitivity, it is *per se* not evident that calculated FRET efficiencies will be equally accurate at such low concentrations. Therefore, we determined, for both samples, the concentration limit for accurately estimating the proximity ratio. As shown in Figure 3C, P-values for the DNA were constant at concentrations above 200 pM, while significant deviations were present below 200 pM. We attribute this deviation to a small signal-to-noise ratio; the signal from the DNA sample is now comparable to the background, causing larger uncertainties in P. Nucleosomal FRET, on the contrary, was constant down to slightly lower concentrations, with significant deviations starting below 100 pM. This reflects the stronger signal in the transfer channel due to FRET.

The low signal-to-noise ratio at pM concentrations also affects the reproducibility of P between sample wells. Figure 3D quantifies the spread in well-wise P-values for sample concentrations in the sub-nanomolar range. For each data set, 25 wells were filled with the same donor-only sample solution. Pseudo P-values were calculated from the signal in the donor channel and the crosstalk of the donor into the transfer channel; therefore, P-values should be constant for all samples and variability dependent upon instrument noise only. Figure 3D shows the standard deviation in P along with an estimation of shot noise based on Poisson photon statistics. The insert figures show the distribution of well-wise P-values for 25 pM, 100 pM and 400 pM respectively. While we observed a sharp distribution at higher sample concentration, P-values at 25 pM are broadly distributed and only crudely approximated by a Gaussian function. The standard deviation is significantly higher at lower sample concentrations. Overall, the variation in P-values is much larger than expected from pure photon statistics, which for the data presented only amounts to 10–15%.of the total variation in P. Intensities in the donor and transfer channel are averaged over several hundred pixels, thus the variation in intensity due to Poisson statistics is minute. The major contribution to uncertainty in P likely arises from experimental error, such as sample handling errors or variations in well-to-well background, rather than from instrumental shot noise.

We conclude that μpsFRET yields consistent and reproducible results for concentrations above 100–150 pM, with the exact sensitivity limit depending on the P-value observed. At smaller concentrations the poor signal-to-noise ratio induces significant deviations in P. We note that for 25 pM sample, the error in P (ΔP  = 0.009) is small compared to the average proximity ratio of a medium FRET sample (P>0.3) but might become important for samples with very low energy transfer.

### c) Acetylation of histone H3 is sufficient to destabilize nucleosome structure

So far, we have demonstrated the potential to measure smFRET at high sample concentration and the ability to probe single molecule relevant conditions efficiently with a bulk FRET assay. We now apply these two techniques to study the effect of histone H3 acetylation on nucleosome structure and stability. This will serve as a model system for the general case of analyzing the unknown effects of a given nucleosome modification. In the last section we shall then verify that both techniques provide comparable estimates of nucleosome stability, thereby validating our approach to combine both techniques to optimize experimental workflow.

For illustration, we tested the role of histone H3 acetylation on nucleosome structure during salt-induced dissociation. Histone H3 was chemically acetylated prior to octamer refolding and nucleosome reconstitution [15]. We first characterized bulk nucleosome stability using μpsFRET to determine relevant conditions for subsequent characterization of sample heterogeneity at the single molecule level.

**Figure 4:**
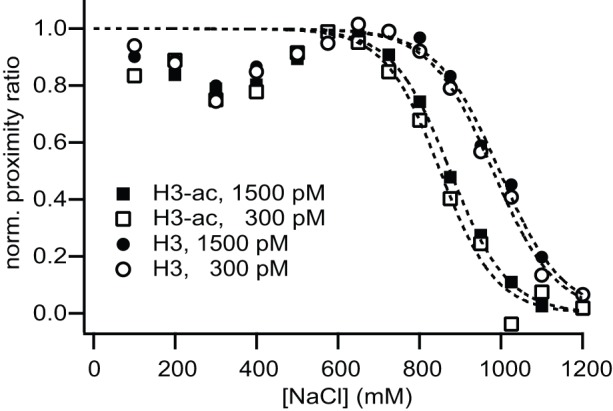
Acetylation of histone H3 decreases nucleosome stability. Salt-dependent proximity ratio at 1.5 nM and 300 pM nucleosome concentration measured with μpsFRET. A loss in P is interpreted as nucleosome dissociation. Salt titration curves were approximated by a sigmoidal function and nucleosome stability was quantified in terms of the c_1/2_ value, the salt concentration at which P is half the maximum observed around 500–600 mM NaCl. Measured c_1/2_ values were (995±20) mM and (980±15) mM for 1.5 nM and 300 pM non-acetylated nucleosomes, while measured c_1/2_-values were 120−130 mM lower for H3-acetylated nucleosomes ((875±10) mM and (850±20) mM for 1.5 nM and 300 pM).

μpsFRET experiments were performed by incubating non-acetylated or H3-acetylated nucleosomes in an array containing different salt (100–1200 mM) and nucleosome concentrations (1.5 nM and 300 pM); reactions were performed in a freshly cleaned and passivated microplate. Samples were incubated at room temperature and scanned after 60 minutes. Each sample was measured in triplicate from which an average P-value was calculated. The average proximity ratio measured for each condition is shown in Figure 4. For all samples we observed a slight decrease in P at lower ionic strength, followed by an increase in P at salt concentrations near 600 mM NaCl. At higher ionic strength nucleosomes dissociated, indicated by a steady decrease in P. All salt titration curves were approximated by a sigmoidal function and nucleosome stability was quantified in terms of the c_1/2_ value, the salt concentration at which P is half the maximum observed around 500–600 mM NaCl. Measured c_1/2_ values were (995±20) mM and (980±15) mM for 1.5 nM and 300 pM non-acetylated nucleosomes, while for H3-acetylated nucleosomes c_1/2_  = (875±10) mM and c_1/2_  =  (850±20) mM for 1.5 nM and 300 pM respectively. This data suggest that the difference between 1.5 nM,and 300 pM nucleosome concentration has a minor effect on stability only; the difference in c_1/2_ is within the error bars for both nucleosome samples. The effect of histone H3 acetylation, however, is more striking, with nucleosomes containing acetylated histone H3 dissociating at significantly lower ionic strength. From these data, we find that dissociation of both acetylated and non-acetylated nucleosomes occurs under intermediate salt concentrations at sub-nanomolar concentrations and that acetylation of histone H3 strongly affects nucleosome stability.

### d) H3-acetylation changes nucleosome structure prior to gross unwrapping

While this bulk data is very useful, it does not tell us whether the observed FRET changes are a result of conformational intermediates or are strictly due to gross unwrapping of DNA. In particular the increase in P-value at salt concentrations around 500–600 mM NaCl is unclear; it could indicate a conformational change in nucleosome structure that precedes gross DNA unwrapping or it could arise from an unexpected increase in nucleosome stability (an increase in the number of FRET active samples). In the first case we shall further ask whether or not acetylation of H3 affects the occurrence of this conformational change.

**Figure 5:**
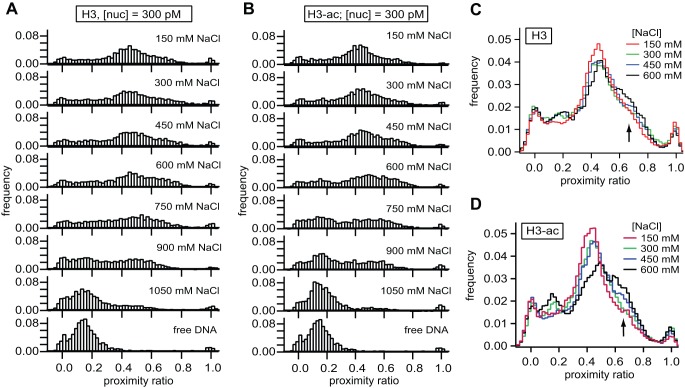
smFRET analysis reveals a conformational transition prior to nucleosome unwrapping. **A,B**) smFRET histograms of non-acetylated and H3-acetylated nucleosomes at various salt concentrations and 300 pM total nucleosome concentration. Above 300 mM NaCl, a fraction of H3-acetylated nucleosomes populates a second conformation with slightly increased proximity ratio compared to non-acetylated nucleosomes, which appear to retain their initial structure. **C, D**) Overlay of histograms for salt concentrations between 150 mM and 600 mM NaCl for non-acetylated (C) and H3-acetylated nucleosomes (D). Data were smoothed once to better visualize the gradual transition of nucleosomes into the high FRET state.

To detail the structural heterogeneity of H3-acetylated and non-acetylated nucleosomes we performed a salt titration experiment using quasi-bulk smFRET. To compare the smFRET data with the plate scanning assay, samples were incubated at 300 pM total nucleosome concentration in the same microplate and under identical buffer conditions as in the bulk experiments. After incubation for 60 minutes, smFRET data were collected for 30 minutes. As shown in Figures 5A and B, at 150 mM NaCl both smFRET distributions look similar, with a major population centered around P = 0.4; we identify this peak with an intact nucleosome complex [20]. As the salt concentration is gradually increased to 600 mM NaCl a redistribution of some nucleosomes to a conformation with increased proximity ratio takes place. This transition is responsible for the observed increase in the average P-value and is promoted by acetylation of histone H3. This transition was accompanied by only a mild increase in free DNA species; the latter increasing substantially only at salt concentrations exceeding 750 mM NaCl. This suggests that the transition to higher FRET primarily occurs at the expense of the initial conformation dominating at low salt. Above 1000 mM NaCl almost all nucleosome samples were dissociated regardless of whether histone H3 was acetylated or not. Figures 5C and D present an overlay of histograms between 150 and 600 mM NaCl to visualize the conformational change in nucleosomes better. Our observation that acetylation of histone H3 promoted this transition is further backed up by a statistical analysis of the FRET distributions (see Section S4 in File S1).

Based on the smFRET distribution alone we cannot assign a specific structure to the high FRET peak, but several observations suggest that this state is a structural intermediate in nucleosome architecture *en route* to dissociation. First, the state has a higher abundance if histone H3 was acetylated; acetylation of histones is known to destabilize the nucleosome and to open nucleosome structure, potentially forming intermediate states during the dissociation pathway. Second, it occurs at lower ionic strength than the loss of FRET due to gross unwrapping of nucleosomes, suggesting a causal relation between this state and subsequent unwrapping. Third, supplemental burst parameter analysis (see Figure S3) excluded aggregated nucleosomes as the origin of the high FRET peak and suggests that the hydrodynamic structure is still similar to that of an intact nucleosome (Figures S3a–c). Finally, quasi-bulk smFRET experiments performed at 2 nM (Figures S3d–f) showed that the high-FRET population is suppressed by the presence of more nucleosomes in solution, which is also known to stabilize nucleosomes.

Taken together, the μpsFRET and smFRET data demonstrate that acetylation of histone H3 significantly destabilizes nucleosomes, but leads to a similar disassembly pathway as non-acetylated histones and promotes the formation of a conformational intermediate during disassembly.

### e) smFRET and μpsFRET yield identical results for nucleosome dissociation

We conclude this article by showing that μpsFRET and smFRET yield the same results on nucleosome dissociation. This is pivotal to the development of a protocol that combines both techniques to optimize experimental workflow. To compare the dissociation data obtained from smFRET with the μpsFRET data we calculated the salt dependence of two parameters: a) the average proximity ratio based on all photons detected from double-labeled single molecules, and b) the relative fraction of FRET-active nucleosomes in the histogram. The first resembles a bulk FRET experiment with the exception that single-labeled species have been removed from the data by restricting our analysis to events with 0<P<0.9. The amount of donor-only species present in smFRET is fairly small and mostly the result of acceptor photodeactivation at high laser intensities used in smFRET; as pointed out in *Materials and Methods*, the amount of single labeled nucleosomes at the time of preparation was less than 5%.

Bulk data derived from smFRET and µpsFRET can vary significantly, if the amount of donor-only or acceptor-only species is no longer small compared to intact double-labeled samples. In such cases, more precise smFRET experiments are needed which allow to separate donor-only species and double-labeled species through alternating laser excitation schemes [34], [35], which probe for the presence and intactness of the acceptor dye. Single labeled pecies were rare in our experiments, so that the residual inclusion of spurious donor-only events did not affect nucleosome stability analysis.

**Figure 6:**
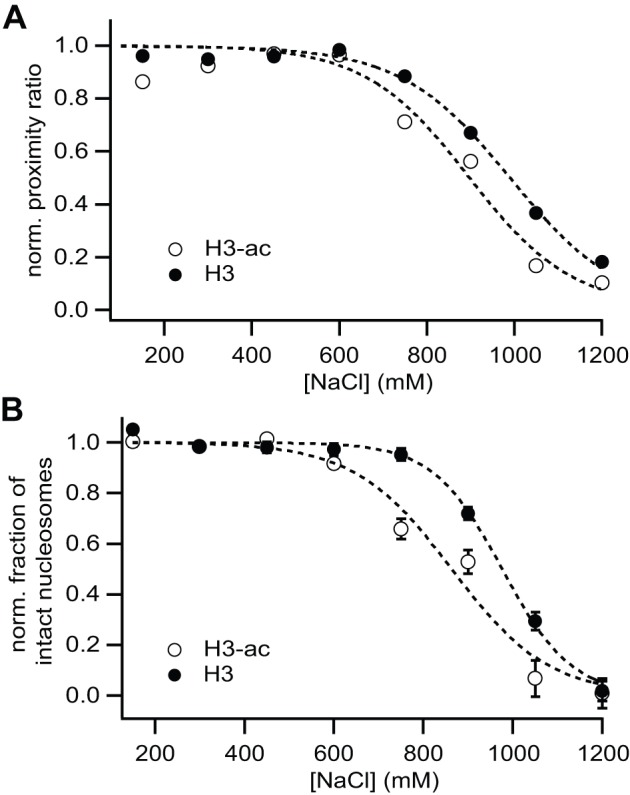
smFRET results on nucleosome stability are consistent to μpsFRET data. (**A**) Average proximity ratio calculated from all photons from double-labeled nucleosomes as a function of salt concentration. Photons from the donor and transfer channel were summed for all detected molecules, except donor-only and acceptor-only species. (**B**) Salt dependence of the fraction of intact nucleosomes in smFRET histograms from Fig. 5. For each histogram, the donor-only and acceptor-only population was excluded from the analysis. The relative fraction of FRET-active molecules (0.25<P<0.9) is plotted against salt concentration. Sigmoidal curves were approximated to the data to extract c_1/2_ values, which are listed in Table 1. After fitting data were normalized to better visualize the difference between non-acetylated and H3-acetylated nucleosomes.

To compare smFRET data with that obtained from μpsFRET, c_1/2_ values were extracted from sigmoidal fits as described above. Figure 6 shows both parameters as a function of salt concentration. After fitting, data were normalized to visually enhance the differences between non-acetylated and H3-acetylated samples. Bulk P-values from the smFRET data showed a similar increase around 600 mM NaCl as those observed in μpsFRET, caused by the transition of some nucleosomes into the high-FRET state. Measured P-values were comparable between both instruments; intensity-averaged P-values im smFRET at 600 mM NaCl were P = 0.44 and P = 0.42 for non-acetylated and H3-acetylated nucleosomes, while P = 0.38 and P = 0.40 were measured in μpsFRET respectively. The relative fraction of FRET-active nucleosomes, on the contrary, did not change significantly at these salt concentrations, confirming that the transition originated mainly from the initial state present at low salt. Table 1 compares the c_1/2_ values calculated for the μpsFRET data with those obtained in smFRET. Bulk averaging of single molecule photons yielded slightly larger estimates of c_1/2_; the difference, however, is still within the fit errors. The effect of histone H3 acetylation is reflected in all analysis schemes. This confirms that μpsFRET and smFRET can both be used to follow nucleosome dissociation with adequate accuracy.

**Table 1:** Comparison of c_1/2_ – values from μpsFRET and smFRET experiments.

	c_1/2_ (mM) , H3	c_1/2_ (mM) , H3-ac
μpsFRET	980±15	850±20
smFRET (bulk P)	990±15	895±35
smFRET (intact fraction)	975±10	865±30

## Discussion

FRET is a sensitive tool for exploring how nucleosome structure and dynamics are modulated by posttranslational modifications, associated factors and DNA sequence. These effects can occur over a large range of sample concentrations; current FRET techniques cannot probe this vast parameter space uniformly. Particularly critical is the range from low nM to a few hundred pM in which neither ensemble nor single molecule FRET works satisfactorily.

**Figure 7:**
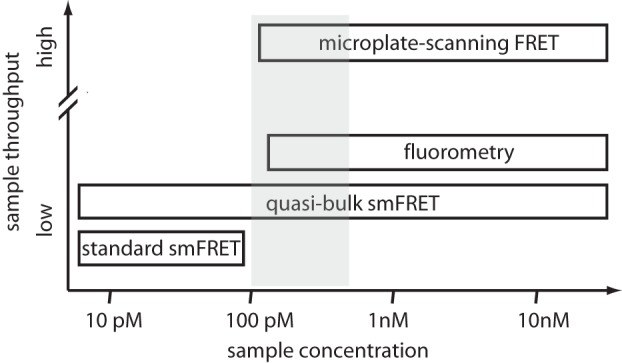
Operational regime of different FRET methods. Conventional smFRET and fluorometric assays are difficult to perform in the intermediate concentration regime (100 pM to 1 nM, shaded area). Quasi-bulk smFRET and microplate-scanning FRET (μpsFRET), on the contrary, allow us to accurately determine FRET efficiencies in this regime and effectively close the gap between single molecule and ensemble FRET spectroscopy. A large range of sample concentrations is now amenable to fast, high throughput estimation of bulk FRET efficiencies (μpsFRET) as well as to a detailed analysis of conformational heterogeneity within the ensemble (quasi-bulk smFRET).

In this work we closed this concentration gap with a scheme that optimizes the workflow for efficient (single molecule) FRET characterization of nucleosomes. We have refined a bulk FRET technique that performs simultaneous FRET spectroscopy on multiple samples and over a wide range of sample concentrations in a 384-well microplate. This ”microplate scanning FRET” (μpsFRET) multiplexes ensemble FRET analysis, consumes significantly less sample than cuvette-based fluorometry and has high sensitivity (100–150 pM detection limit) and a large dynamic range, from 100 pM to, in principle, several µM and more (Figure 7). Samples can be stored for several days to follow the evolution of hundreds of samples over time, and be easily recovered for later analysis via gel electrophoresis. The upper concentration limit to μpsFRET depends on the nature of the sample; concentrations at which (unwanted) sample aggregation occurs are no longer useful for μpsFRET. Signal strength also poses problems at very high concentrations, driving the detector into saturation. This can be circumvented, however, by reducing the amount of labeled species and adding unlabeled nucleosomes in μpsFRET samples.

Complementary to μpsFRET we used a “quasi-bulk smFRET” approach to detect single nucleosome heterogeneity over an extended range of sample concentrations. Concentrations that are amenable to smFRET are typically below the dissociation constant of most DNA-protein interactions, with the exception of few cases, such as lac-repressor binding to DNA [33] or nucleosome formation on the “Widom 601” sequence. Quasi-bulk smFRET probes a subset of labeled species that is present in a bulk concentration of unlabeled nucleosomes and which is representative for the whole ensemble. We demonstrated the usefulness of this concept with a comparison of nucleosomes reconstituted on two different sequences; the resulting histogram reflected the stability of the total nucleosome ensemble and not only that of the labeled subset.

Adding unlabeled species to raise the total nucleosome concentration is efficient in FRET assays, only when acceptor and donor fluorophore are on the same subunit of the nucleosome, in our case the DNA. Subunits can exchange between labeled and unlabeled complexes and there is a high probability for the formation of single labeled species if different subunits were labeled. The resulting loss in FRET signal is no longer indicative of disassembly but results from subunit exchange. In such cases multicolor smFRET setups are beneficial, which allow to discriminate between single labeled and double labeled complexes through alternating laser excitation [34]–[36]. Other approaches to stabilize samples at sub-nM concentrations have been discussed, such as confinement in a gel matrix [37] or encapsulation of molecules in picolitre containers, such as liposomes [38] or water-in-oil droplets [39], [40]. Confinement prevents diffusional loss of subunits and increases the local concentration, but does not allow simple sample extraction for downstream processing and suffers from a large surface-to-volume ratio, which might affect the dynamic properties of the confined molecule. In our assay unlabeled nucleosomes offer the best way to generate well defined concentrations that are amenable to bulk as well as single molecule FRET experiments.

Using μpsFRET and smFRET in combination, we optimized the experimental workflow for nucleosome analysis: instead of mapping an extended parameter space using time-consuming smFRET, we first narrow down the range of relevant conditions using μpsFRET. For this limited set of conditions single molecule experiments can then probe the structural changes induced by specific nucleosome modifications, thereby optimizing the usage of instrument time. We highlight this concept by measuring changes in nucleosome structure and stability upon acetylation of histone H3. In bulk, H3-acetylation rendered nucleosomes more susceptible to salt-induced dissociation. More importantly, an increase in bulk P was observed at lower salt concentrations than those at which the loss of FRET due to nucleosome disassembly occurred. At these conditions smFRET experiments revealed significant changes in the conformational heterogeneity of nucleosomes, a conformational transition of some nucleosomes into a state with higher FRET. This conformation appears to be a first intermediate in the pathway of nucleosome destabilization, which occurs prior to gross unwrapping of the nucleosome. Since formation of this intermediate is promoted by acetylation of histone H3, its structure may play an important role in nucleosome accessibility. Acetylation has been shown to facilitate the passage of DNA polymerase through chromatin during transcription [41]. We speculate that nucleosomes in this intermediate state might lack one or both histone H2A-H2B dimers. It has been shown by others and ourselves that during salt-induced disassembly the H2A-H2B dimer is released first [17], [18], [21], [42] and that H2A-H2B dimers can exchange between nucleosomes at much higher rates than the tetramer [43]. The structural transition was observed at salt concentrations similar to those required for the opening of the (H2A-H2B):(H3–H4)_2_ interface [21]. Suppression of this intermediate state occurred at higher nucleosome concentration, which further corroborates our speculation. We previously demonstrated H2A–H2B dimer exchange between nucleosomes under sub-nanomolar concentrations and elevated ionic strength (see supplemental information in ref. [21]). An excess of unlabeled nucleosomes could provide a pool of free dimers that can shuttle between open nucleosome conformations and promote refolding into intact nucleosomes through octamer reassembly (main population with P≈0.4). It will be interesting to determine which specific acetylated residue(s) promote this conformational transition and whether other protein factors may utilize or modulate the prominence of this state.

Our results highlight the benefit of combining μpsFRET and smFRET to characterize the effects of histone modifications on nucleosome structure and accessibility. μpsFRET rapidly detected the presence of a conformational transition around 500 mM NaCl in one experiment, but was unable to provide structural insights. It did, however, help to identify relevant conditions for more efficient, subsequent smFRET characterization, . Based on μpsFRET, subsequent smFRET experiments can be targeted to either the dissociation process itself ([NaCl]  = 700–1000 mM) or to conformational changes prior to dissociation ([NaCl] <700 mM). In this work we presented smFRET data from both regimes for demonstration.

A successful combination of bulk and single molecule assays for nucleosome stability is only possible if both methods yield comparable results of nucleosome stability. Here, we demonstrated that μpsFRET and smFRET provide consistent estimates of nucleosome stability; smFRET-based c_1/2_ values of the change in both average FRET as well as the fraction of FRET-active molecules agreed with changes measured in our microplate assay. Absolute P-values were also similar for both methods, showing that our experiments were performed with comparable settings. Although absolute FRET efficiencies were not important in our stability assay, more general applications, however, will require μpsFRET and smFRET to yield comparable FRET efficiencies if used on the same sample. Section S6 in File S1 and Figure S4 demonstrate the ability of our methods to accurately determine absolute FRET efficiencies from a model system of short DNA standards.

We finally note that bulk FRET of diluted nucleosome samples has also been analyzed with confocal microscopy to determine the effect of DNA sequence on nucleosome stability [44]. These experiments have provided valuable insight into the effect of label position on the outcome of the experiment; yet they only sampled one condition at a time with limited accuracy at pM concentrations. Our μpsFRET approach will be beneficial for these types of experiments since it provides enhanced sample throughput with comparable, if not better, signal quality. Furthermore, one could imagine testing other parameters with this method, such as fluorescence anisotropy.

A broad range of concentrations can now be accurately analyzed in bulk and on the single molecule level, which offers great benefits to efficient FRET experiments on nucleosomes and other protein-DNA complexes. Much has yet to be learned about the structural and dynamic changes imposed on nucleosomes by posttranslational modifications, histone variants, or nucleosome modifying enzymes. We envision our assay as being a useful framework to probe heterogeneous FRET changes in macromolecular systems.

## Supporting Information

Figure S1 **Similarity of P histograms for different acquisition times.** **a–d)** 50 pM of acetylated nucleosomes were incubated in a low salt buffer and two smFRET histograms were acquired for t_ac_  = 2, 10, 20 and 40 minutes each. Burst selection thresholds were set to >70 photons and <100 µs interphoton time to enhance the contrast between subpopulations. Detected number of bursts: 128 and 111 (t_ac_  = 2 min); 496 and 521 (t_ac_  = 10 min); 984 and 1077 (t_ac_  = 20 min); 2061 and 2053 (t_ac_  = 40 min). Histograms are shown in black and grey in the upper panel of each subfigure. The bottom panels show the bin-wise difference between the normalized histograms. Only coarse-grained distributions were obtained after two minutes, with large deviations between both recordings. Longer acquisition times result in smooth, reproducible histograms. For 20 and 40 minutes data acquisition no significant differences between the two histograms were observed.
(EPS)

Figure S2 **Passivation strategies for FRET experiments.** A Typhoon scan imaging the surface of a 384-well multiplate, showing the adsorption of fluorescently labeled nucleosomes to the bottom surface. Prior to incubation two wells were silanized with Sigmacote™ and a small amount of the surfactant Nonidet P40 was added to the buffer solution where noted. An untreated, empty well is shown for reference. Best passivation was achieved using a combination of surface silanization and addition of surfactant into the buffer solution.
(EPS)

Figure S3 **Selective burst parameter analysis of nucleosomes at 400 mM NaCl.** **a)** Definition of the subpopulations considered for subspecies analysis; “no FRET” (NF: 0.05< P<0.25), “medium FRET” (MF; 0.25< P<0.55) and “high FRET” (HF; 0.55< P<0.9). **b, c)** Burst duration versus burst size distributions for H3 non-acetylated (b) and H3-acetylated (c) nucleosomes. **d–f)** Quasi-bulk smFRET of H3-acetylated nucleosomes at 300 pM (red curves) and 2 nM nucleosome concentration (black curves) for 450 mM (d), 600 mM (e) and 750 mM NaCl (f). Histograms are normalized to equal height of the medium FRET population at P  =  0.4 to visualize the relative change in high-FRET abundance as the nucleosome concentration is increased.
(EPS)

Figure S4 **Absolute quantification of FRET efficiencies from DNA standards. a)** μpsFRET grey-scale images from 1 nM solutions of buffer and DNA samples (donor-only, acceptor-only, FRET11 and FRET22). For each sample four wells were filled with the same solution and imaged on the Typhoon. Voltages were set to 600 V for the donor channel and 660 V for the transfer and acceptor channel. **b)** smFRET histograms of donor-only, FRET22 and FRET11 DNA. Samples were diluted to 40 pM, smFRET data were taken for 10 minutes (donor-only sample) or 20 minutes (FRET22 and FRET11). Histograms are shown prior to correction for direct acceptor excitation. Only a small amount of donor-only species is present in the FRET-active samples. **c)** The helical model that was used to estimate interfluorophore distances in FRET22 and FRET11. The interdye distance is given by D  =  (L^2^ + R^2^)^1/2^, where the axial displacement L and radial component R are given as L [nm]  = 0.34*ΔN and R  = 2(l+r)sin(α/2), where α [°]  = 34.1*ΔN+180 is the angle enclosing both dye linkers. ΔN is the separation between both fluorophores in base pairs, l the length of the flexible linkers and r the radius of double stranded DNA.
(EPS)

Table S1 **Statistical distribution momenta for smFRET histograms between 150 mM and 600 mM NaCl.**
(DOCX)

Table S2 **Comparison of absolute FRET efficiencies from smFRET, μpsFRET and the helical model of DNA.**
(DOCX)

File S1 Section 1: Experimental section. Section 2: Duration and sample throughput of confocal smFRET experiments. Section 3: Passivation of 384-well multiplates for FRET experiments. Section 4: Analysis of distribution momenta for smFRET histograms. Section 5: Subspecies analysis of histone H3-acetylated nucleosomes. Section 6: Comparison of absolute FRET efficiencies from μpsFRET and smFRET.
(DOC)
