# Supplementary material for: Closing the Gap between Single Molecule and Bulk FRET Analysis of Nucleosomes
Source: PLoS One. 2013 Apr 18;8(4):e57018. doi: 10.1371/journal.pone.0057018 (PMC3630217; doi:10.1371/journal.pone.0057018)
Supplement: Table S1 — Statistical distribution momenta for smFRET histograms between 150 mM and 600 mM NaCl. (DOCX) [file pone.0057018.s005.docx]

**Table S1: Statistical distribution momenta for smFRET histograms between 150 mM and 600 mM NaCl.**

|  | **H3** | | | **H3-ac** | | |
| --- | --- | --- | --- | --- | --- | --- |
| **[NaCl]** | **Average** | **Std. dev.** | **Skewness** | **Average** | **Std. dev.** | **Skewness** |
| 150 mM | 0.498 | 0.137 | 0.506 | 0.476 | 0.135 | 0.699 |
| 300 mM | 0.514 | 0.152 | 0.423 | 0.491 | 0.138 | 0.559 |
| 450 mM | 0.515 | 0.144 | 0.376 | 0.504 | 0.139 | 0.409 |
| 600 mM | 0.525 | 0.146 | 0.225 | 0.525 | 0.142 | 0.173 |
