# Supplementary material for: Closing the Gap between Single Molecule and Bulk FRET Analysis of Nucleosomes
Source: PLoS One. 2013 Apr 18;8(4):e57018. doi: 10.1371/journal.pone.0057018 (PMC3630217; doi:10.1371/journal.pone.0057018)
Supplement: Table S2 — Comparison of absolute FRET efficiencies from smFRET, μpsFRET and the helical model of DNA. (DOCX) [file pone.0057018.s006.docx]

**Table S2: Comparison of absolute FRET efficiencies from smFRET, μpsFRET and the helical model of DNA.**

| **construct** | **E (smFRET)** | **E (μpsFRET)** | **E (model)** |
| --- | --- | --- | --- |
| **FRET11** | 0.58±0.01 | 0.62±0.01 | 0.64±0.02 |
| **FRET22** | 0.11±0.01 | 0.10 ± 0.01 | 0.09±0.01 |
