# Supplementary material for: Closing the Gap between Single Molecule and Bulk FRET Analysis of Nucleosomes
Source: PLoS One. 2013 Apr 18;8(4):e57018. doi: 10.1371/journal.pone.0057018 (PMC3630217; doi:10.1371/journal.pone.0057018)
Supplement: File S1 — Section 1: Experimental section. Section 2: Duration and sample throughput of confocal smFRET experiments. Section 3: Passivation of 384-well multiplates for FRET experiments. Section 4: Analysis of distribution momenta for smFRET histograms. Section 5: Subspecies analysis of histone H3-acetylated nucleosomes. Section 6: Comparison of absolute FRET efficiencies from μpsFRET and smFRET. (DOC) [file pone.0057018.s007.doc]

**Section 1: Experimental section**

a) Preparation of DNA-based FRET standards

For the comparison of absolute FRET efficiencies between both instruments we prepared DNA oligonucleotides of 31 base pairs in length (sequence: 5’-CCGCTCAATTGGTCCAGGCACGTGTCAGATG-3’). Alexa 488 was attached to the first T-base at position 5 from the 5’-end via amino-C6 linker. Alexa 594 was attached on the complementary DNA strand at distances 11 bp and 22 bp from the donor dye position respectively. Single stranded DNA’s were mixed and heated up to 95 °C for 10 minutes and slowly cooled down to 20°C for annealing. Hybridization quality was checked on a native 20% polyacrylamide gel.

b) Confocal single molecule setup

Experiments were performed on a home-built system, in which an inverted microscope was equipped with confocal excitation and detection optics. Fig. 1C shows a schematic view of the setup. The donor was selectively excited with the 488 nm line of a continuous-wave Ar^+^/Kr^+^-laser (Melles Griot). The beam diameter was reduced to underfill the back focal aperture of a high NA objective lens to enlarge the focus size. Laser-induced emission of the dyes was detected in epifluorescence mode; the fluorescence passed a dichroic mirror (505DRLP) and a 100 µm pinhole located in a conjugated image plane of the objective. The pinhole defined an effective focal volume of about 1 fL by rejecting any out-of-focus light. The transmitted fluorescence was split into two spectral windows defined by a replaceable filter unit that housed a dichroic beam splitter (580DRLP) and appropriate interference filters (donor channel: 520DF40, transfer channel: 610ALP, all from Omega Optical). A lens in front of each detector focused the fluorescence onto the active area of an avalanche photodiode (SPAD-AQ-14, Perkin-Elmer). The single photon signal from the detectors was read out by a TCSPC board (TimeHarp200, Picoquant GmbH). An analysis software developed in our group discriminated single molecule events against background based on the time difference between two successive photons. These interphoton times were first smoothed to reduce the effect of noise in the signal (filter size 15 photons). Bursts were usually defined as groups of photons with interphoton times smaller than 120 microseconds. The exact thresholds were set by visual inspection of the filtered interphoton time trace in the analysis software.

For each registered event, several burst parameters were calculated, including burst duration, burst size and burstwise photon rate (burst size divided by burst duration). These parameters were used to discriminate further against multi-particle events [29] and to characterize further any subpopulations in the smFRET histogram.

c) Intensity correction for background, crosstalk and direct excitation

In µpsFRET and smFRET, P is estimated from measured donor and acceptor raw intensities I^0^_D_ and I^0^_T_. In general these contain unwanted contributions from background (B_D_ and B_T_), donor crosstalk into the acceptor channel (α_DT_) and direct excitation of the acceptor dye (f_dir_), which need to be corrected for,

$\begin{matrix} I_{T}=\left(I_{T}^{0}-B_{T}\right)-\alpha_{DT}\left(I_{D}^{0}-B_{D}\right)-f_{dir} \\ I_{D}=\left(I_{D}^{0}-B_{D}\right) \end{matrix}$. (S1)

Here, we briefly summarize how to derive the appropriate correction factors experimentally.

Background

In µpsFRET the average background per sample well, B_D_ and B_T_, is measured directly in one or more separate wells containing pure buffer solution.

In smFRET, first an average background count rate is measured for the buffer solution, yielding rates b_D_ and b_T_ as photons per millisecond. For the i-th single molecule event b_D_ and b_T_ are then multiplied with the duration of the event, d^i^:

$B_{D,T}^{i}=b_{D,T}\cdot d^{i}$. (S2)

Crosstalk of donor emission into the acceptor channel

In µpsFRET one or more wells are filled with a donor-only sample and measured in parallel to double-labeled FRET samples. From the intensities in the donor and acceptor channel upon excitation with 488 nm, (I_Dex_^Dem^)_D-only_ and (I_Dex_^Aem^)_D-only_, we obtain the crosstalk factor as

$\alpha_{DT}=\frac{(I_{Dex}^{Aem}-B_{T})_{D-only}}{(I_{Dex}^{Dem}-B_{D})_{D-only}}$. (S3)

In smFRET the donor-only sample is measured for 5-10 minutes to build a histogram of the proximity ratio P, equation 2. After background subtraction, the peak value in the P histogram, P_D-only_, yields the crosstalk factor as

$\alpha_{DT}=\left(\frac{1}{1/P_{D-only}-1}\right)^{-1}$. (S4)

Alternatively, α_DT_ can be directly estimated from a donor-only single molecule histogram of the intensity ratio of transfer channel to donor channel.

Direct excitation of the acceptor

The protocol for direct excitation (f_dir_) correction differs slightly between µpsFRET and smFRET. In µpsFRET f_dir_ is determined with an independent measurement of an acceptor-only sample. All samples are probed with two lasers; a laser that selectively excites the acceptor, yielding acceptor intensities (I_Aex_^Aem^)_A-only_ and (I_Aex_^Aem^)_FRET_, and the laser that preferentially excites the donor dye, yielding intensities (I_Dex_^Aem^)_A-only_ and (I_Dex_^Aem^)_FRET_ for the acceptor-only and FRET samples respectively.

After background correction the only contribution to (I_Dex_^Aem^)_A-only_ comes from direct acceptor excitation and we can define a ratio

$S_{A}=\frac{\left(I_{Dex}^{Aem}-B_{T}\right)_{A-only}}{\left(I_{Aex}^{Aem}-B_{A}\right)_{A-only}}$. (S5)

S_A_ is expected to be the same for the acceptor molecules in the FRET-active sample and the acceptor-only sample. Based on the measured intensity (I_Aex_^Aem^)_FRET_, we thus obtain the contribution of direct excitation in the actual FRET sample as

$f_{dir}=S_{A}\cdot\left(I_{Aex}^{Aem}-B_{A}\right)_{FRET}$. (S6)

In one-color smFRET f_dir_ is estimated as described in [36]. Based on the number of detected photons in the donor channel, I_D_, an estimated detection factor γ, energy transfer E and extinction coefficients ε_D_ and ε_A_ of donor and acceptor at 488 nm, the amount of photons due to direct acceptor excitation is given by

$f_{dir}=\frac{\varepsilon_{A}}{\varepsilon_{D}}\cdot\frac{I_{D}}{(1-E)\gamma}.$ (S7)

**Section 2: Duration and sample throughput of confocal smFRET experiments**

To quantify the minimum acquisition time required for accurate smFRET experiments we compared the reproducibility of P histograms for identical samples that were measured for different acquisition times. 50 pM of acetylated nucleosomes were diluted in smFRET buffer containing 20 mM NaCl. smFRET histograms were acquired for 2, 10, 20 and 40 minutes; each experiment was done twice; the resulting histograms were superimposed to visualize the spread in data between both successive recordings.

As shown in Figure S1, a coarse-grained histogram was already obtained after 2 minutes: the distributions, however, only discriminated two major subpopulations. Significant variations between both histograms were present due to the low number of molecules detected. The variation between successive recordings diminished for longer acquisition times, when more single molecule events contributed to the histogram. For acquisition times of 20 and 40 minutes, no significant differences between the two recordings were observed. In our setup, under typical conditions we required a minimum acquisition time of 15-20 minutes for reliable smFRET histograms.

The sample throughput can be increased by carefully optimizing the threshold criteria for burst recognition and increasing the number of photons detected from a single molecule. The latter can be achieved by slowing down the Brownian motion of the molecule, e.g. by increasing the viscosity of the buffer via addition of substances like glycerol, sucrose or Ficoll™, or by enlarging the focus volume. Even under optimized conditions, however, smFRET experiments will still require several minutes to yield accurate P distributions, severely limiting sample throughput in smFRET.

**Section 3: Passivation of 384-well multiplates for FRET experiments**

Avoidance of sample adsorption to the chamber walls is crucial for the success of FRET experiments at nanomolar nucleosome concentrations. To find an optimal passivation strategy a diluted solution of fluorescently labeled nucleosomes was placed in 384-well multiplates that were either treated with Sigmacote™, Nonidet P40, or a combination of both. Figure S2 shows a grey scale image of the resulting sample adsorption. In the case of an untreated glass surface extensive adsorption of nucleosomes was observed. Silanization of the surface with Sigmacote™ resulted in lower sample adsorption, yet the observed signal was still stronger than if Nonidet P40 was added into the buffer. Sample adsorption was absent when the glass surface was treated with Sigmacote™ and a small amount of Nonidet P40 (0.01% w/w) was added into the buffer solution.

**Section 4: Analysis of distribution momenta for smFRET histograms**

To provide additional evidence that acetylation of histone H3 promotes transition of nucleosomes into the high FRET state that was observed in smFRET experiments (Fig. 5C and 5D) we calculated the the average value, standard deviation and skewness of the P-value distribution in the salt range 150 mM – 600 mM NaCl.. To exclude donor-only, acceptor-only and FRET-inactive species, we restricted the analysis to events with 0.25 < P < 0.9. For this subset, average P, standard deviation and skewness were calculated using the built-in statistics function in IGOR Pro. Changes in average P signify either a redistribution between states or a uniform shift of all P-values. The skewness, on the contrary, is a direct measure of the asymmetry of the distribution and independent of the average P. It can distinguish between a simple shift of all P-values (no change in skewness) and a redistribution between states. A transition between two populations, e.g. medium FRET and high FRET, would increase the average P-value and reduce the asymmetry of the distribution until both populations become equal. The calculated statistical parameters are listed in Table S1 for non-acetylated and H3-acetylated nucleosomes. Both, the increase in average proximity ratio as well as the decrease in skewness are indicative of a conformational transition upon salt increase. Both parameters show a stronger variation in H3-acetylated samples than in non-acetylated samples, which supports our findings that acetylation of H3 promotes formation of the high FRET conformation. The standard deviation does not change significantly as a function of salt concentration.

**Section 5: Subspecies analysis of histone H3-acetylated nucleosomes**

To shed further light on the nature of the high-FRET peak, we analyzed various single burst parameters including burst duration, burst size and the burstwise photon rate. As shown in Figure S3a single molecule events were grouped into three subpopulations; a “no FRET” population containing DNA and dissociated nucleosomes (NF, 0.05 < P < 0.25), a “medium FRET” population that is centered around P = 0.4 (MF: 0.25 < P < 0.55) and a “high FRET” population centered around P = 0.75 (HF, 0.55 < P < 0.9). Data from H3-acetylated nucleosomes were compared to data from the non-acetylated sample at 450 mM NaCl. Panels b and c show a scatter plot of burst size versus burst duration for both samples; no significant difference between the subspecies was observed. Events in the noFRET population show a slight increase in events with larger burst duration due to the increase in diffusion time of a free DNA / unfolded nucleosome [21]. The similarity between both species suggests that the high FRET peak is not an aggregate of partially dissociated nucleosomes but rather similar in shape and positioning to a compact nucleosome.

Figures S3d-f show quasi-bulk smFRET experiments on H3-acetylated nucleosomes at 2 nM and 300 pM sample concentrations for 450 mM, 600 mM and 750 mM NaCl. Samples were incubated for 60 minutes prior to smFRET experiments. These salt conditions were chosen, because the high FRET state was significantly populated at 300 pM nucleosome concentration. Histograms are rescaled to equal peak height of the medium FRET population to better visualize the change in the relative fraction of the high FRET population. Addition of unlabeled nucleosomes significantly reduced the relative amount of nucleosomes found in the high FRET state. We attribute this to the stabilization of the intact nucleosome conformation at higher sample concentration, possibly due to exchange of histone components, such as the H2A-H2B dimers, between open conformations, enabling refolding of some partially dissociated nucleosomes into an intact conformation.

**Section 6: Comparison of absolute FRET efficiencies from μpsFRET and smFRET**

In this section we show that absolute FRET efficiencies measured with both instruments are accurate and comparable. The energy transfer, E, is calculated from the measured proximity ratio P and the detection factor γ via

$E=\frac{1}{\gamma(1/P-1)+1}$, (S8)

where γ = (η_A_^A^ϕ_A_)/(η_D_^D^ϕ_D_), with η_A_^A^ (η_D_^D^) being the detection efficiency for acceptor (donor) fluorescence in their respective detection channels. ϕ_A_ and ϕ_D_ are the quantum yields of acceptor and donor fluorophore. The detection factor of a home-built confocal system can be estimated from the optical components and the spectral emission signature of the dyes. This is not straightforward for a commercial system such as the Typhoon scanner, where the design of the intermediate optical system might be proprietary. Therefore, we used a strategy, where experimentally both instruments were adjusted to the same detection factor. The proximity ratios for identical samples was then compared and the absolute value of γ determined from the confocal data, Finally, experimentally determined FRET efficiencies were compared to those predicted from a helical model of double-stranded DNA.

To do so we rewrite the detection factor in terms of the absorption cross sections and molecular brightness values of the fluorophores:

$\gamma=\frac{\eta_{A}^{A}\varphi_{A}}{\eta_{D}^{D}\varphi_{D}}=\frac{I_{A}^{A}/(I_{0}\sigma_{A}\varphi_{A}n_{A})}{I_{D}^{D}/(I_{0}\sigma_{D}\varphi_{D}n_{D})}\cdot\frac{\varphi_{A}}{\varphi_{D}}=\frac{(I_{A}^{A}/n_{A})}{(I_{D}^{D}/n_{D})}\cdot\frac{\sigma_{D}}{\sigma_{A}}$. (S9)

I_0_ denotes the laser excitation intensity, σ_A_ and σ_D_ are the absorption cross sections of acceptor and donor at the excitation wavelength (488 nm). n_A_ and n_D_ are the number of donor and acceptor molecules in the observation region. The intensity per particle, (I_A_^A^/n_A_) or (I_D_^D^/n_D_), is the molecular brightness of the molecule. In the confocal system the molecular brightness can be determined from the autocorrelation function of the single labeled species. In μpsFRET these quantities are not directly available. γ only depends on the ratio of both parameters, however, and both instruments will have the same γ, if the ratio of the intensities I_A_^A^/I_D_^D^ from a given set of single-labeled samples is the same in both instruments and if the ratio σ_D_/σ_A_ does not differ. The latter assumption is usually valid for a narrow band excitation around the same wavelength in both instruments.

31 bp long DNA constructs, labeled with Alexa 488 and Alexa 594 served as FRET standards. Two FRET-active samples were used, denoted as “FRET11” and “FRET22”, with both dyes being separated by 11 and 22 base pairs respectively. DNA sequence and dye positions are given in section S1. Nominal solutions of 1 nM of all samples (donor-only, acceptor-only, FRET11 and FRET22) were prepared in TE buffer, supplemented with 100 mM NaCl, 0.01% Nonidet P40 and 0.5 mM ascorbic acid.

Samples were first measured in μpsFRET with voltages set to 600 V for the donor and 660 V for the transfer and acceptor channel, yielding an (unknown) detection factor γ_Typ_. Figure S6a shows grey scale images of the intensity signal from all three detection channels. Donor-only and acceptor-only samples yielded an intensity ratio of (I_A_^A^/I_D_^D^)_Typ_ = 0.0272; measured proximity ratios for the FRET-active samples were P = 0.17±0.01 for FRET22 and P = 0.74±0.01 for FRET11.

We next used the single-labeled samples to adjust the detection factor of the confocal instrument, γ_Conf_. Using deliberately detuned detection (D3) [14] we changed the detection efficiencies to obtain an intensity ratio (I_A_^A^/I_D_^D^)_Conf_ = 0.0270; we thus expect γ_Conf_ ≈ γ_Typ_. For smFRET samples were then diluted to 40 pM concentration and measured for 10 minutes (donor-only) or 20 minutes (FRET11 and FRET22). No acceptor-only histogram was taken in smFRET. Laser power was set to 50 μW to minimize artifacts due to photodestruction. All data were corrected for background, donor crosstalk into the acceptor channel and direct acceptor excitation as described in section S1. Histograms of smFRET data prior to the correction for direct acceptor excitation are shown in Figure S6b, demonstrating that only a small amount of donor-only events were present in the double-labeled samples. After correcting for f_dir_, a single Gaussian was approximated to the intact sample peak to yield center P-values of P = 0.18±0.01 for FRET22 and P = 0.70±0.01 for FRET11 respectively.

Next, γ was determined from the ratio of the extinction coefficients and the molecular brightness of the donor-only and acceptor-only sample at 488 nm, see eq. S9. The latter were determined using FCS to be I_A_^A^/n_A_ = 6.1 and I_D_^D^/n_D_ = 84.2 at the laser intensity used for smFRET. The extinction coefficients of both samples were independently measured using bulk fluorometry, where we obtained a ratio ε_D_/ε_A_ = 23.5, which we assume to be equal to the ratio of the absorption cross sections, σ_D_/σ_A_, Combined, these numbers give an estimated detection factor of γ = 1.71. Based on eq. S8, measured proximity ratios then translated into energy transfer values E_exp,Typ_^FRET22^ = 0.10±0.01 and E_exp,Typ_^FRET11^ = 0.62±0.01 for μpsFRET and E_exp,Conf_^FRET22^ = 0.11±0.01 and E_exp,Conf_^FRET11^ = 0.58±0.01 for smFRET.

We finally compared these experimental FRET values to those predicted from a helical DNA model as described by Clegg et al. [7]. Figure S6c shows a cartoon of the model and its relevant parameters. Axial displacement L and radial component R are given as L [nm] = 0.34*ΔN and R [nm] = 2(l+r)sin(α/2), where the angle enclosing both dyes is defined as α [°] = 34.1*ΔN+180. ΔN is the separation between both fluorophores in number of base pairs. A length of l = (0.70±0.05) nm was assumed for the flexible linkers, as well as a radius of r = 1 nm for the DNA helix. The interdye distance then follows as D = (L^2^ + R^2^)^1/2^. Estimated interdye distances for FRET 22 and FRET11 were D^FRET22^ = (8.17±0.04) nm and D^FRET11^ = (5.03±0.07) nm. Taking a Förster radius of R_0_=5.56 nm, which we previously determined for both fluorophores attached to nucleosomal DNA [20], these translate into expected FRET efficiencies around E_theo_^FRET22^ = 0.09±0.01 and F_theo_^FRET11^ = 0.64±0.02.

For both constructs the data obtained in μpsFRET agree well with the prediction from the DNA model, demonstrating its potential to measure absolute FRET efficiencies. Between both instruments the data for FRET22 are in good agreement with each other, while experimental E-values for FRET11 showed a ≈ 7% discrepancy, which we attribute to slight differences in the actual γ between both FRET samples. Adjustment of the detection system was done using single-labeled samples derived from the FRET22 construct. Thus it is not surprising that the data from FRET22 agreed well between both instruments. Using independent bulk measurements we found that the local environment of the acceptor dye in the two constructs is not identical, showing a small (≈ 3 nm) shift in absorption and emission spectra (data not shown). Thus, in the confocal setup, the acceptor detection efficiency for FRET11 was about 8% smaller than for FRET22, while the spectral shift had almost no effect in the Typhoon setup. Here, the emission filter is centered on the emission maximum and a small spectral shift should not affect the overlap between emission spectrum and filter transmission. An only 10% smaller detection factor would change E_exp,Conf_^FRET11^ to 0.60±0.01, a value that is closer to the energy transfer measured in µpsFRET.

In summary our data show that, given the various sources of error associated with FRET experiments with one-color excitation, both methods are able to determine absolute FRET efficiencies with sufficient accuracy.
